# Trends in Diabetic Ketoacidosis Hospitalizations and In-Hospital Mortality — United States, 2000–2014

**DOI:** 10.15585/mmwr.mm6712a3

**Published:** 2018-03-30

**Authors:** Stephen R. Benoit, Yan Zhang, Linda S. Geiss, Edward W. Gregg, Ann Albright

**Affiliations:** 1 National Center for Chronic Disease Prevention and Health Promotion, Division of Diabetes Translation, CDC.

Diabetes is a common chronic condition and as of 2015, approximately 30 million persons in the United States had diabetes (23 million with diagnosed and 7 million with undiagnosed) (*1*). Diabetic ketoacidosis (DKA) is a life-threatening but preventable complication of diabetes characterized by uncontrolled hyperglycemia (>250 mg/dL), metabolic acidosis, and increased ketone concentration that occurs most frequently in persons with type 1 diabetes (*2*). CDC’s United States Diabetes Surveillance System* (USDSS) indicated an increase in hospitalization rates for DKA during 2009–2014, most notably in persons aged <45 years. To explore this finding, 2000–2014 data from the Agency for Healthcare Research and Quality’s National Inpatient Sample (NIS)^†^ were assembled to calculate trends in DKA hospitalization rates and in-hospital case-fatality rates. Overall, age-adjusted DKA hospitalization rates decreased slightly from 2000 to 2009, then reversed direction, steadily increasing from 2009 to 2014 at an average annual rate of 6.3%. In-hospital case-fatality rates declined consistently during the study period from 1.1% to 0.4%. Better understanding the causes of this increasing trend in DKA hospitalizations and decreasing trend in in-hospital case-fatality through further exploration using multiple data sources will facilitate the targeting of prevention efforts.

NIS is a nationally representative sample of hospital discharges, corresponding to >35 million hospitalizations annually. Discharges with a first-listed, primary *International Classification of Diseases, Ninth Revision, Clinical Modification* (ICD-9-CM) code of 250.1 (diabetes with ketoacidosis) were considered DKA hospitalizations (*1*,*3*). To calculate DKA hospitalization rates among persons with diabetes, the civilian, noninstitutionalized population with diabetes was estimated using corresponding years of data from CDC’s National Health Interview Survey (NHIS).^§^ Because diabetes type was not collected in NHIS, rates were not stratified by disease type. In-hospital case-fatality rate was defined as the proportion of persons hospitalized for DKA who died in the hospital.

DKA rates were age-adjusted using four age groups (<45, 45–64, 65–74, and ≥75 years) from the 2000 U.S. Census. Joinpoint regression models,^¶^ which use permutation tests to identify points where linear trends change significantly in direction or magnitude, were used to analyze trends in DKA hospitalization and case-fatality rates, stratified by age group and sex, allowing for a maximum of two joinpoints. In the final model, each trend segment was described by an annual percent change, and the trend for each period was tested to determine whether the slope was significantly different from zero. A p-value of <0.05 was considered statistically significant. The analysis accounted for the complex sampling designs of NIS and NHIS.

From 2000 to 2009, the age-adjusted rate of DKA hospitalizations among persons with diabetes fluctuated but declined at an average annual rate of 1.1% (Figure) (Table 1). During 2009–2014, however, the rate increased 54.9%, from 19.5 to 30.2 per 1,000 persons, at an average annual rate of 6.3%. The reversal in trend was apparent across all age groups and both sexes. Rates were highest in persons aged <45 years (44.3 per 1,000 in 2014) and lowest in persons aged ≥65 years (<2.0 per 1,000) (Table 1).

**FIGURE:**
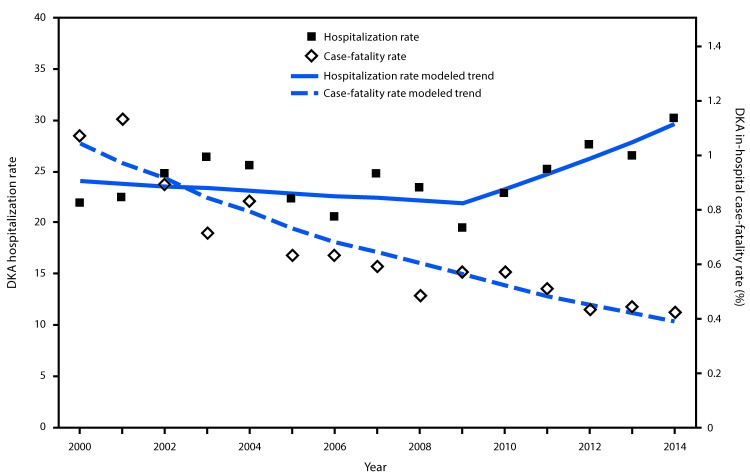
Age-adjusted diabetic ketoacidosis hospitalization rate per 1,000 persons with diabetes and in-hospital case-fatality rate — United States, 2000–2014* **Abbreviation:** DKA = diabetic ketoacidosis. * Symbols indicate observed points; lines indicate modeled trends. All modeled trend lines were significant at a p-value of <0.05.

**TABLE 1:** Diabetic ketoacidosis hospitalization rates per 1,000 persons with diabetes, overall and by age group and sex — National Inpatient Sample and National Health Interview Survey, United States, 2000–2014

Characteristic	Year 2000 (N = 12,052,000*) Rate (95% CI)	First joinpoint year	First joinpoint (N = 20,667,000*^,†^) Rate (95% CI)	Year 2014 (N = 21,953,000*) Rate (95% CI)	APC (95% CI)	
					Period 1^§^	Period 2^§^
**Total DKA, no.^¶^**	**101,621**	**2009**	**141,704**	**188,950**	**NA**	**NA**
Total rate**	**21.9 (18.9 to 24.9)**	**2009**	**19.5 (16.8 to 22.3)**	**30.2 (26.4 to 34.0)**	**-1.1 (**-**1.8 to **-**0.3)**	**6.3 (4.6 to 8.0)**
**Age group (yrs)**						
<45	31.7 (27.1 to 36.3)	2009	28.5 (24.3 to 32.7)	44.3 (38.5 to 50.0)	-1.0 (-1.8 to -0.2)	6.2 (4.5 to 8.0)
45–64	4.6 (4.1 to 5.1)	2009	3.7 (3.3 to 4.0)	5.2 (4.8 to 5.5)	-1.5 (-1.9 to -1.1)	6.7 (5.8 to 7.5)
65–74	1.5 (1.3 to 1.7)	2007	1.0 (0.9 to 1.2)	1.6 (1.5 to 1.8)	-5.2 (-5.8 to -4.6)	6.4 (5.9 to 6.9)
≥75	1.6 (1.3 to 1.8)	2007	0.9 (0.7 to 1.0)	1.4 (1.2 to 1.5)	-9.3 (-10.3 to -8.3)	6.0 (4.9 to 7.0)
Sex**						
Male	23.9 (18.7 to 29.1)	2009	18.6 (14.6 to 22.5)	30.8 (25.3 to 36.2)	-2.5 (-3.4 to -1.6)	8.0 (6.1 to 10.0)
Female	20.2 (16.8 to 23.6)	2009	20.5 (16.6 to 24.3)	29.6 (24.8 to 34.3)	0.1 (-0.7 to 0.9)	5.1 (3.1 to 7.0)

**Abbreviations:** APC = annual percent change; CI = confidence interval; DKA = diabetic ketoacidosis; NA = not applicable.

* Data rounded to the nearest thousand.

^†^ Population with diabetes in 2009.

^§^ Period 1 is from 2000 to first joinpoint year; period 2 is from first joinpoint year to 2014.

^¶^ Estimated number of DKA hospitalizations in the indicated years.

** Age adjusted to the 2000 U.S. Census using the four age groups listed in the table.

In-hospital case-fatality rates declined during 2000–2014 at an annual average rate of 6.8% (from 1.1% to 0.4% [63.6% decline overall]); no joinpoints were found (Figure) (Table 2). The declining rates were seen across all age groups and both sexes. Although the highest case-fatality rates were observed among persons aged ≥75 years, this group experienced the largest absolute decrease across the entire period.

**TABLE 2:** Diabetic ketoacidosis in-hospital case-fatality rates, overall and by age group and sex — National Inpatient Sample, United States, 2000–2014

Characteristic	Year 2000 (N = 101,621) % (95% CI)	First joinpoint year	First joinpoint (N = 141,704*) % (95% CI)	Year 2014 (N = 188,950) % (95% CI)	APC (95% CI)	
					Period 1^†^	Period 2^†^
**No. of deaths^§^**	800	2009	611	620	NA	NA
**Total^¶^**	**1.1 (0.9 to 1.2)**	—**	—**	**0.4 (0.4** to **0.5)**	**-6.8 (**-**7.1 to **-**6.4)**	—**
**Age group (yrs)**						
<45	0.3 (0.2 to 0.4)	2007	0.1 (0.1 to 0.2)	0.1 (0.1 to 0.1)	-13.1 (-14.6 to -11.5)	-3.3 (-5.2 to -1.2)
45–64	1.0 (0.7 to 1.2)	—**	—**	0.5 (0.3 to 0.6)	-5.4 (-6.1 to -4.7)	—**
65–74	3.4 (2.2 to 4.6)	2007	1.5 (0.6 to 2.3)	1.4 (0.8 to 1.9)	-10.0 (-13.6 to -6.4)	-2.4 (-6.8 to 2.3)
≥75	7.2 (5.2 to 9.2)	—**	—**	2.6 (1.6 to 3.6)	-7.0 (-7.7 to -6.3)	—**
**Sex^¶^**						
Male	1.2 (0.9 to 1.5)	—**	—**	0.5 (0.4 to 0.6)	-6.9 (-7.3 to -6.4)	—**
Female	1.0 (0.8 to 1.2)	—**	—**	0.4 (0.3 to 0.5)	-6.6 (-7.1 to -6.1)	—**

**Abbreviations:** APC = annual percent change; CI = confidence interval; DKA = diabetic ketoacidosis; NA = not applicable.

* Number of DKA hospitalizations in 2009.

^†^ Period 1 is from 2000 to first joinpoint year (or 2014 if no joinpoint); period 2 is from first joinpoint year to 2014.

^§^ Estimated number of in-hospital deaths.

^¶^ Age adjusted to the 2000 U.S. Census using the four age groups listed in the table.

** No joinpoints were found.

## Discussion

Although DKA hospitalization rates among persons with diabetes declined slightly from 2000 to 2009, this trend reversed, with rates increasing 54.9% from 2009 to 2014. From 2009 to 2014 all age groups experienced an increase of ≥6.0% annually in DKA hospitalization rates, with highest rates among persons aged <45 years. This increase in DKA hospitalization rates is concerning because DKA is a life-threatening but avoidable complication of diabetes. Despite the increase in DKA hospitalization rates, however, in-hospital mortality among persons with DKA consistently decreased over the study period. Identification of factors contributing to the increase in hospitalizations for DKA might help target prevention efforts.

Although DKA is more common among persons with type 1 diabetes, it also occurs among persons with type 2 diabetes (*2*). DKA can be the initial sign of unrecognized type 1 or type 2 diabetes; however, it occurs more frequently in persons with established disease (*4*). Two studies among youths found either stable or decreasing rates of DKA at the time of diagnosis of diabetes, suggesting that younger persons with established disease and poor glucose control might be the group contributing most to the increase in DKA hospitalization rates among persons aged <45 years (*5*,*6*). However, whether DKA is occurring at the time of diagnosis or among persons with established disease in adults is unknown.

The causes of the increase in DKA hospitalization rates are not clear, but several possible explanations include the following: changes in case definition, new medications that might increase the risk for DKA, and higher admission rates because of lower thresholds for hospitalization (i.e., admission of persons with less serious disease). Although the American Diabetes Association definition of DKA has not changed over the years, the most recent 2009 publication described a “euglycemic DKA” type characterized by metabolic acidosis and increased total body ketone concentration, but with glucose levels ≤250 mg/dL, occurring in approximately 10% of patients with DKA (*7*). Euglycemic DKA hospitalizations might have resulted in an increase in the number of hospitalized patients classified as having DKA, but NIS does not have laboratory data to corroborate this hypothesis. This hypothesis assumes that DKA case definitions are uniformly applied in clinical practice, which in a call for standardization of diagnostic criteria for DKA was demonstrated to be unlikely (*8*).

Sodium-glucose cotransporter 2 (SGLT2) inhibitors, a class of prescription medications used to treat type 2 diabetes, were approved in March 2013. In December 2015, the Food and Drug Administration added a label to SGLT2 inhibitors warning that these medications might increase the risk for DKA. Because SGLT2 inhibitors were only recently approved, and DKA rates increased before their introduction, they are likely not a major contributor to the increasing DKA trend but do remain an ongoing concern for future events. More recent higher admission rates for less severe cases of DKA could also explain an increased trend in DKA admissions. Although no evidence that this occurred exists, analyzing emergency department data might help confirm or refute this hypothesis.

The causes of the decrease in DKA in-hospital mortality are also not clear. Better understanding of the pathophysiology of DKA and adoption of DKA treatment guidelines, both of which might have led to better management and treatment, have been proposed as reasons for declines in DKA in-hospital mortality (*2*). Another possibility is that hospital admission of less severe cases has resulted in higher admission rates and contributed to the lower in-hospital case-fatality rates over time.

In the early 2000s, an increase in DKA cases among persons with obesity and type 2 diabetes was reported (*9*). These patients had impaired insulin levels but lacked typical autoimmune markers of type 1 disease, and their beta-cell function recovered quickly after treatment. This disease type, named ketosis-prone type 2 diabetes, has features of both type 1 and type 2 disease. Other variants of diabetes have been described, but no evidence that rates of these disease variants are increasing and thus contributing to the increased DKA hospitalization rates could be found (*10*).

In 2014, the DKA hospitalization rate among persons with diabetes aged <45 years was approximately 27 times the rate among persons aged ≥65 years. Therefore, efforts to understand factors contributing to the increase in hospitalizations for DKA should consider the demographic and clinical characteristics of youths and young adults. Information from studies among these groups might help determine whether factors such as symptom recognition, adherence to therapy, self-management skills, access to care, or cost of treatment should be a focus of DKA prevention strategies.

The findings in this report are subject to at least four limitations. First, NIS does not include federal hospitals, which would lead to an underestimate of the total number of DKA hospitalizations; however, NIS represents approximately 96% of the U.S. population. Second, although in-hospital DKA case-fatality rates declined, mortality rates at home or in the emergency department setting were not investigated. Third, the DKA case definition used in this analysis was based on an ICD-9-CM code from the hospital discharge record that could not be validated. Misclassification might have occurred leading to over- or underestimation of hospitalization rates. However, misclassification caused by changing coding practices over time is unlikely. Finally, results were not stratified by diabetes type, and persons with type 1 diabetes are at particularly high risk for DKA. 

DKA hospitalizations in the United States have increased among all age groups, with the highest rates among persons aged <45 years. Although the continued decline in in-hospital DKA mortality is encouraging, further work might help identify populations at risk. Evidence-based, targeted prevention measures, such as diabetes self-management education and support might help reverse the trend in this potentially life-threatening but avoidable complication of diabetes.

**Summary**

**What is already known about this topic?**

Diabetic ketoacidosis (DKA) is a life-threatening complication of diabetes, a disease that affects approximately 30 million persons in the United States. DKA is more common among persons with type 1 diabetes.

**What is added by this report?**

After a slight decline during 2000–2009, hospitalizations for DKA increased in the United States during 2009–2014 among all age groups and were highest among persons aged <45 years. Concurrently, in-hospital case-fatality rates among persons with DKA consistently decreased from 2000 to 2014.

**What are the implications for public health practice?**

DKA is a life-threatening but avoidable complication of diabetes. Prevention measures, such as diabetes self-management education, might help reverse the increasing trend in DKA, especially in persons aged <45 years who have the highest DKA rates.

## Footnotes

* https://www.cdc.gov/diabetes/data.

† https://www.hcup-us.ahrq.gov/nisoverview.jsp.

§ https://www.cdc.gov/nchs/nhis/methods.htm.

¶ https://surveillance.cancer.gov/joinpoint.
